# Effects of a Long-Term Monitored Exercise Program on Aerobic Fitness in a Small Group of Children with Cystic Fibrosis

**DOI:** 10.3390/ijerph19137923

**Published:** 2022-06-28

**Authors:** Wolfgang Gruber, Florian Stehling, Christopher Blosch, Stefanie Dillenhoefer, Margarete Olivier, Cordula Koerner-Rettberg, Sivagurunathan Sutharsan, Uwe Mellies, Christian Taube, Matthias Welsner

**Affiliations:** 1 Pediatric Pulmonology and Sleep Medicine, Cystic Fibrosis Center, Children’s Hospital, University of Duisburg-Essen, 45117 Essen, Germany; florian.stehling@uk-essen.de (F.S.); christopher.blosch@uk-essen.de (C.B.); margarete.olivier@uk-essen.de (M.O.); u.mellies@prof-mellies.de (U.M.); 2 Institute of Human Nutrition and Food Science, Christian Albrechts University Kiel, 24105 Kiel, Germany; 3 Department of Pediatric Pulmonology, University Children’s Hospital, Ruhr University Bochum, 44791 Bochum, Germany; stefanie.dillenhoefer@ruhr-uni-bochum.de; 4 Children’s Hospital Marienhospital Wesel, 46483 Wesel, Germany; cordula.koerner-rettberg@prohomine.de; 5 Department of Pulmonary Medicine, University Hospital Essen—Ruhrlandklinik, Adult Cystic Fibrosis Center, University of Duisburg-Essen, 45239 Essen, Germany; sivagurunathan.sutharsan@rlk.uk-essen.de (S.S.); christian.taube@rlk.uk-essen.de (C.T.); matthas.welsner@rlk.uk-essen.de (M.W.)

**Keywords:** cystic fibrosis, children, aerobic fitness, VO2peak, long-term exercise program, physical activity

## Abstract

Background: The aim of this study was to investigate the effects of a monitored exercise program on aerobic fitness in children with cystic fibrosis (CF). Methods: Six children (2f/4m) with ages ranging from 6 to 14 years (11.3 ± 3.3 years.) and a mean ppFEV1 102.5 ± 13.5% pred. participated in the partially monitored 12-month exercise program. VO2peak and Wpeak were used as parameters of aerobic fitness. Incremental Cardio-Pulmonary Exercise Tests (CPETs) were performed before the program began (T1), after 6 months (T3) of monitoring, and after a further 6 months (T4) without monitoring. Habitual physical activity (HPA) was assessed with accelerometry. Results: The values of VO2peak and Wpeak improved slightly from T1 to T3 (*p* > 0.05), without a further increase after monitoring was stopped (T4). However, the VO2peak and Wpeak values were higher after monitoring was stopped compared to at T1. The exercise program with and without monitoring (*p* > 0.05) had no or only a slight effect on the FEV1 values, steps/day, and the intensity of HPA. Conclusions: Monitoring seems to facilitate the achievement of beneficial effects on physical fitness in CF children. For that reason, continuous individual exercise monitoring programs that involve close contact with an exercise therapist should be provided to maintain long-term motivation and participation in physical activities and sport activities during leisure time.

## 1. Introduction

Cystic fibrosis (CF) is a genetic disease that is caused by mutations in the cystic fibrosis transmembrane conductance regulator (CFTR) gene [1]. Impaired CFTR function affects the regulation of chloride ion transport in epithelial cells in tissues in various organ systems, especially in the lungs, intestinal system, pancreas, and sweat glands [2]. Due to abnormal CFTR protein function, abnormally thick mucus is produced, leading to inflammation in the lungs, recurrent infections, progressive lung disease, and the insufficiency of the exocrine pancreas [1,2]. Advancements in management and treatment have led to increased life expectancy, and today, survival is expected into the fifth decade [3]. The treatment of CF requires a complex and multidisciplinary regime consisting of pharmacological treatment, dietary interventions, physiotherapy, physical activity, and exercise [4,5].

Exercise programs have been demonstrated to have beneficial effects on exercise capacity, lung function, bone density, aspects of motor performance, and quality of life in people with CF (pwCF) regardless of the severity of the disease [5,6,7,8,9,10,11,12,13]. Furthermore, a higher exercise capacity, expressed as VO2peak, has been associated with a better prognosis [14,15,16]. These effects were observed in short-term exercise programs lasting from 6 to 12 weeks, as well as in long-term exercise programs with durations from 12 to 36 months [5,6,7,8,9,10,12,13].

In adult pwCF, no significant changes were noted between the training and the control group in parameters related to the 6MWT after 12 weeks of supervised exercise training [17]. Significant improvements in muscle strength were only found in adults with CF after a supervised exercise program, whereas lung function remained unchanged [17].

VO2peak values improved after a six-month, partially supervised exercise program in adolescents and adults with CF who participated in three training sessions per week [8,9]. The interventions included aerobic exercise training and/or strength training. When supervision was stopped, a decrease was seen in FEV1 and VO2peak values after 12 months, with a subsequent improvement in the VO2peak values after another 12 months. Habitual physical activity (HPA) with vigorous intensity only changed to a minor extent over the entire period [8].

There are a limited number of studies investigating the effects of supervised aerobic exercise training in children with CF. As already described in adults with CF, exercise programs also have a positive impact on physical and psychological health in children with CF.

Urquhart et al. reported an increase in exercise capacity after a 12-month supervised exercise program with twice-weekly contact with a physiotherapist. Assessments of exercise capacity were conducted using the Modified Shuttle Test (MST), and a significant walking distance in MST was noticed at the end of the program [18].

After a supervised, short-term, in-hospital program in adult pwCF, beneficial effects on VO2peak and FEV1 values were found in the aerobic training group, whereas in the resistance training group, the FEV1 level improved, but not the VO2peak level [19]. Aerobic training intensity was set at 70% of peak heart rate with five session per week, each of 30-min duration [19].

In a recently published study in children with CF, significant increases were found in peak workload (Wpeak), coordination and strength/endurance values [10]. However, when the supervision was stopped, there was a decrease in the Wpeak values, with a small change in parameters of coordination, power, and strength.

With regard to beneficial effects on exercise capacity, it has been shown that greater improvements in parameters could be achieved in supervised compared to unsupervised programs [5]. In general, supervised exercise programs were either hospital-based, home-based, or a combination of both, in which children, adolescents, or adults participated. The participants had close contact with exercise specialists. This close contact seemed to be a facilitator for better adherence to participation in sports and exercise, resulting in a greater increase in physical performance [8,9,10]. 

To date, most of the studies that have included a partially supervised exercise program have evaluated the changes in adolescents and adults with CF with respect to VO2peak as a parameter of exercise capacity.

Less is known about the effects on physical fitness in children with CF after a partially monitored, long-term exercise program, especially when the monitoring ends.

To the best of our knowledge, no study has investigated the long-term effects of a partially monitored exercise program on VO2peak values in children with CF in the outpatient setting.

Thus, the present study aimed to evaluate the effects of a partially monitored, 6-month exercise program on VO2peak values in children with CF. Moreover, the second aim of the study was to investigate the long-term effects on VO2peak values after the end of the monitoring period. We hypothesized that VO2peak values would increase significantly after the 6-month monitoring period, with no further increase or a slight decrease in the 6 months after the end of the intervention. Furthermore, we assumed that habitual physical activity would increase during the monitoring period and remain constant in the following 6 months without monitoring.

## 2. Materials and Methods

In total, 79 children ≥ 6 years were asked to participate in the partially monitored exercise program, which was part of the CFmobil exercise project. CFmobil was carried out at the Christiane Herzog Centrum Ruhr (CHCR), a cooperation of three CF centers (University Children’s Hospital Essen, the University Children’s Hospital Bochum, and the Ruhrlandklinik Essen) located in the Ruhr area in the west of Germany.

The CFmobil project was initiated to establish sport and exercise as additional components of CF therapy to further improve patients’ care. Therefore, the exercise program was offered to all patients, and information was given during the regular hospital visits at the CF center.

The diagnosis of CF was made by at least two pathologic sweat tests (sweat chloride > 60 mmol/L) and/or by the presence of two CF mutations. Exclusion criteria were defined as inadequately treated diabetes mellitus or musculoskeletal problems that did not allow continuous exercise training.

**Figure 1:**
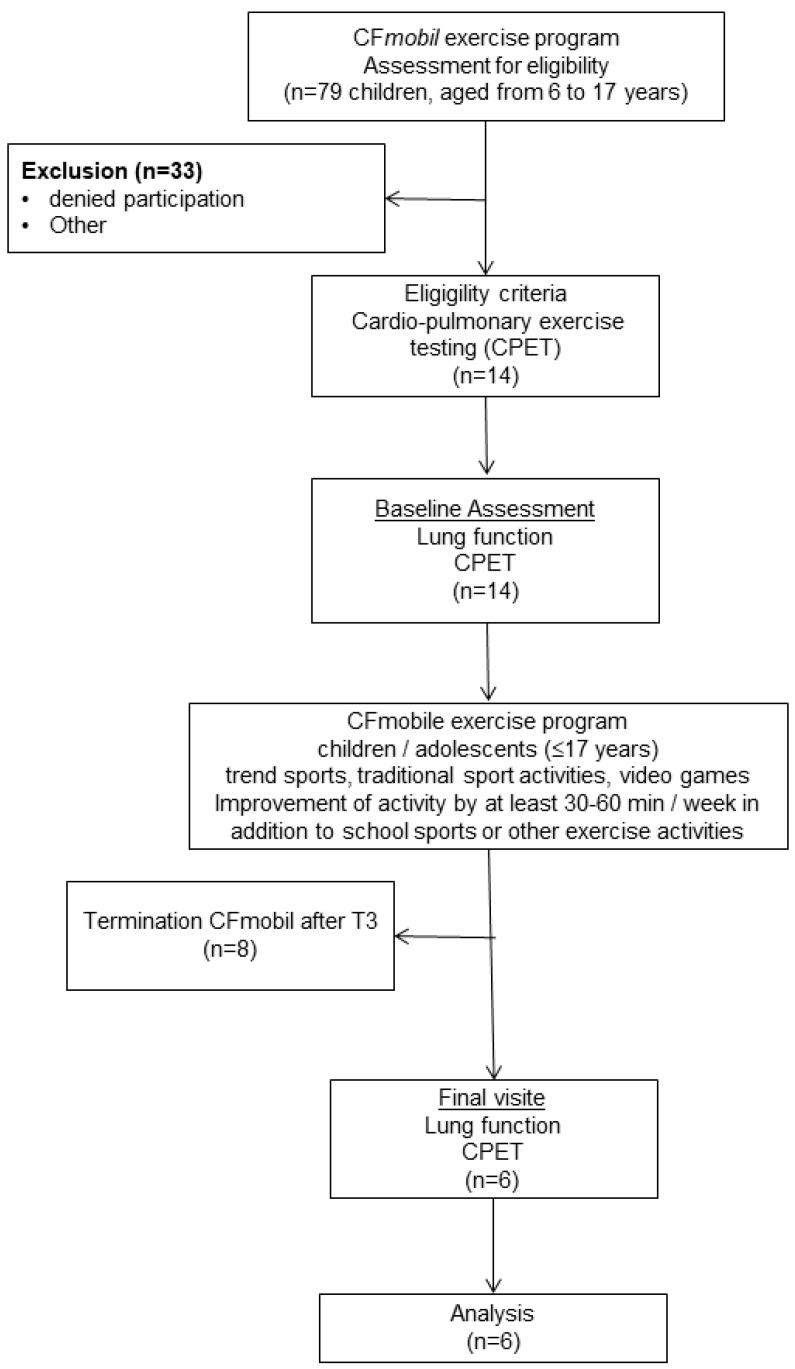
Selection flowchart of participants.

The Cardio-Pulmonary Exercise Testing (CPET) could only be carried out at the University Children’s Hospital in Bochum. Therefore, only n = 14 clinically stable children with CF were eligible to participate in the partially monitored exercise program (Figure 1). All of the children and their caregivers agreed to participate and voluntarily took part in the tests and the exercise program, n = 8 children terminated the exercise program after stopping the period of monitoring (T3), and n = 6 children finished the 12-month exercise program (Figure 1).

The study was approved by the local ethics committees of the University Hospital Essen (14-6117-Bo) and University Hospital Bochum (13-5314), and is listed in Clinical Trials (NCT03518697). The study included children and adolescents aged 6–17 years who had a confirmed diagnosis of CF by at least two pathologic sweat tests and/or by the presence of two defining CF mutations. All of the patients were willing to participate in and to comply with the research project procedure, and written informed consent was given by either one of their parents or a guardian.

### 2.1. Study Design

**Figure 2:**
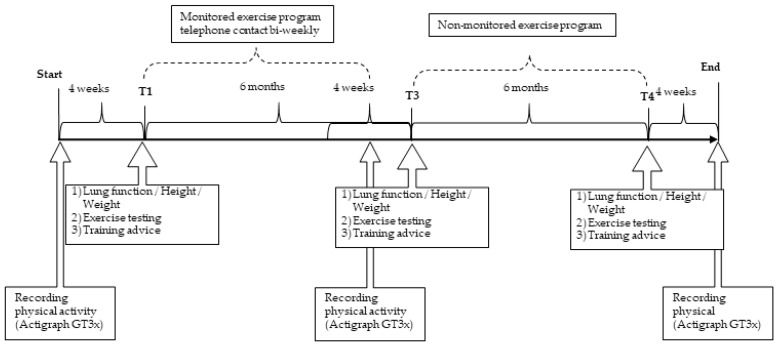
Timeline of CFmobile exercise program.

The study was designed as a pre-experimental study one-group pre-test–post-test intervention study [20]. Anthropometric and pulmonary function data, physical performance data, and habitual physical activity data were collected at three different time points: baseline = T1, at the end of the monitored exercise program after six months (T3), and after further six months without monitoring (T4). Overall, the training program was planned to last 12 months (Figure 2).

The individualized, partially monitored home exercise program was created together with the children and their parents. In the development of the exercise program, the children’s interests, inclinations, exercise capacity, disease-related limitations, and environmental conditions (e.g., place of residence, accessibility of training facilities, etc.) were considered

For this reason, a great variety of physical activities were offered and presented to our participants, for example, trend sports, traditional sport activities, video games (Wii sports^®^ or Microsoft Xbox^®^ with a Kinect device), aerobic exercise, strength training, and games and activities to improve motor/skill performance.

The intensity of the aerobic exercise training was set to 70–80% of the patient’s peak heart rate, or to 90% and 95% of the Ventilatory Anaerobic Threshold (VT2), assessed using CPET [21].

The recommendation for all of the children was to improve their physical activities by exercising at least 30 min daily, in addition to other physical activities [22]. The children should perform aerobic exercise 2 to 3 times a week, and were instructed to include strength training with their own body weight 2 × per week, with the level of intensity mentioned above representing moderate-to-vigorous (3–6 METs) or vigorous intensity (≥6 METs) [22,23]. School sport lessons were calculated as one exercise session/week.

The exercise training program was created together with the sport and exercise therapist. A very important aspect in the joint development of the program was that, in addition to improving aerobic endurance and strength endurance of the major muscle groups, the children had fun while performing it. The aim was to motivate the children to participate in physical activities together with their parents.

Throughout the first 6 months of training, there was constant biweekly telephone contact between the participants or, in younger children, their parents and the sports and exercise scientists.

The telephone calls served to discuss possible problems with the implementation of the exercise program, to discuss motivational or volitional problems, and to look for appropriate solutions, but also to highlight successes. In the non-monitored period, the telephone calls were stopped. In urgent cases, such as disease-related problems, patients or their parents were able to contact the exercise therapist or the physician.

In addition, the patients and their parents had contact during the regular clinic visits every 3 months. After 6 months of the exercise program, monitoring was stopped. In case of clinical problems, the children or their parents were able contact the exercise scientist or supervising physician. The patients kept a diary and listed all of the activities they participated in alone or together with their parents. This diary was the basis of the discussions in telephone interviews and during regular visits to the hospital.

### 2.2. Testing

At baseline (T1) and after 6 months (T3) and 12 months (T4), the lung function, physical fitness, height, and bodyweight of all of the participants were recorded (Figure 2).

An electronic flat scale (seca 861, seca, Hamburg, Germany) was used to assess bodyweight (kg). Height was determined to the nearest 0.1 cm with a telescopic measuring rod (seca 202, seca Hamburg, Germany). Body mass index (BMI) was calculated (kg/m^2^).

Lung function parameters, which were forced expiratory volume in the first second in % of predicted (ppFEV1) and forced vital capacity (ppFVC in % of predicted), were measured using standard spirometric techniques (Jaeger^®^ MasterScreen Body, Vyaire Medical, Hoechberg, Germany) according to ATS guidelines [24].

CPET was performed on an electro-magnetically braked cycle ergometer (ergoselect 200, ergoline, Bitz, Germany) in an upright position. Gas exchange and ventilatory measures were recorded breath by breath (Vyntus^TM^ CPX, Vyaire Medical, Hoechberg, Germany).

After a period of rest (3 min) and after a 3 min phase of unloaded cycling, the work rate was increased every minute by 10–20 W (Godfrey protocol) depending on the patient’s height and physical fitness [25,26]. The participants were encouraged to make a maximal effort, and the test was continued until the subject could no longer maintain a pedaling cadence of 60 rpm, or SpO2 was >85%. To specify the Ventilatory Anaerobic Threshold (VAT), the excess carbon dioxide method (ExCO2), and the modified V-Slope method were used [27]. Heart rate (HR) was measured continuously using a 12 lead ECG.

VO2peak (mL/min/kg) and peak workload (Wpeak in W/kg) were defined as the highest value achieved before the test was terminated. Peak gas exchange and peak ventilatory measures were specified as the highest value during the final 30 s before stopping the test. The percentages of values predicted were determined from the reference equations of Godfrey for workload, and of Orenstein for oxygen uptake [28,29].

Habitual physical activity (HPA) was assessed with a wActiSleep-BT Monitor (Actigraph Corp., Pensacola, FL, United States) over a period of 4 weeks. Physical-activity-related parameters were steps/day and the intensity of HPA expressed as metabolic equivalents (METs, light (<3 METs/min/day), moderate (3–5.9 METs/min/day), and vigorous (≥6 METs/min/day)). A sedentary time period was defined as an intensity of <1.5 METs/min/day [30]. All noted data were averaged over the entire wearing period.

### 2.3. Statistics

All data are displayed as the mean ± standard deviation (SD). Descriptive statistics were calculated, and data were checked for normal distribution (Shapiro–Wilk test). The effects of the exercise program were analyzed using the Friedman two-way Analysis of Variance (ANOVA) by Ranks Test. In the case of significance for the factor “training”, a Wilcoxon’s signed rank test was applied for within-group comparison. Statistical analyses were performed using SPSS 22.0 (SPSS, Inc., Chicago, IL, USA). Significance was set at the 0.05 level for all of the tests.

## 3. Results

### 3.1. Anthropometric Characteristics and Lung Function

Out of a total of 14 children, 6 children completed the partially monitored, 12-month exercise program. Statistically significant improvements were found for height and ppFEV1 values. Height improved significantly (χ^2^ = 9.652, *p* = 0.008) from the baseline (T1) to the end of the program (T4), whereas ppFEV1 values remained almost constant from T1 to T3, followed by a decrease at T4 (χ^2^ = 6.333, *p* = 0.042). All the other anthropometric parameters remained nearly unchanged from T1 to T4 (weight χ^2^ = 4.261, *p* = 0.119, BMI χ^2^ = 0.933, *p* = 0.627) (Table 1).

### 3.2. Habitual Physical Activity

All of the parameters of HPA expressed as steps/day and intensity only changed to a minor extent over the course of the program. A slight increase in steps/day (+5.2%, χ^2^ = 1.600, *p* = 0.449) and HPA with moderate-to-vigorous intensity (+8.7%, χ^2^ = 2.632, *p* = 0.268) could be seen at the end of the monitored period, which, however, decreased again in the following 6 months (−10.3%) after monitoring was stopped. At the end of the monitored exercise program, there was a small, non-significant increase by +5.9% (χ^2^ = 0.737, *p* = 0.692) in PA with light intensity, with a simultaneous decrease in time spent engaging in sedentary activities (−11.8%, χ^2^ = 0.737, *p* = 0.692). However, in the following 6 months, the level of PA with light intensity decreased further, but an increase in the time spent being sedentary was observed (Table 1).

### 3.3. Exercise Capacity

**Figure 3:**
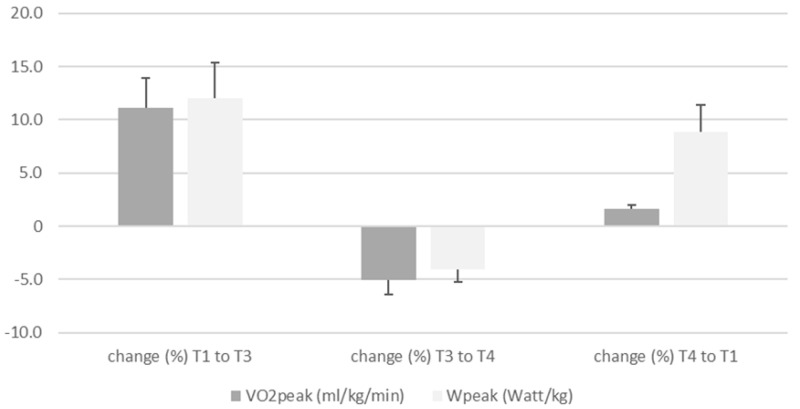
Increase in CPET parameters in percent over the course of the 12months partially monitored exercise program.

**Figure 4:**
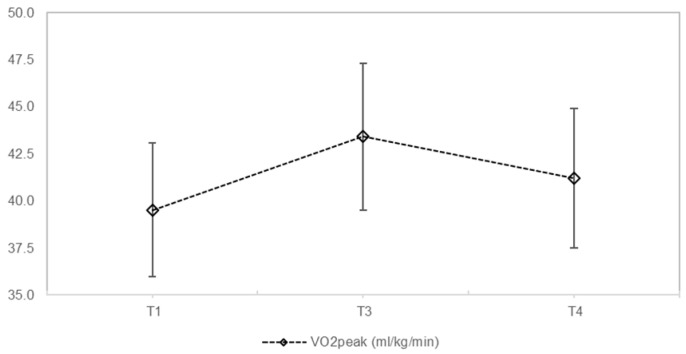
In crease of VO2peak over the course of the 12months partially monitored exercise program.

Improvements in VO2peak (+11.3%, (χ^2^ = 1.600, *p* = 0.449) and VO2 values in %pred. (+7.2%; (χ^2^ = 2.211, *p* = 0.331) were observed after the monitored exercise training program, followed by a decrease in the following 6 months, respectively. However, both values for VO2 (relative and percentage) were higher than the baseline values at the beginning of the training period (T1) (Table 2, Figure 2). Wpeak (Watt/kg) values were found to improve by +10.3% (χ^2^ = 2.666, *p* = 0.264) and by +12% (χ^2^ = 2.311, *p* = 0.311), expressed as Wpeak %pred., but the changes were not significant (*p* > 0.05) from T1 to T3, and remained almost constant from T3 to T4 (Wpeak in W/kg by −3.1 and Wpeak in %pred. by −3.0%, *p* > 0.05) (Table 2, Figure 3). Despite the small decreases from T3 to T4 in VO2peak and Wpeak values, both parameters were still higher at T4 than at the beginning of the exercise training program (Figure 4).

The increase in VO2peak values by almost 11.3% (Figure 3) from T1 to T3 was comparable to the increase in Wpeak values (12.0%), but from T3 to T4, a slightly higher decrease in VO2 values of 5.3% could be noted (Wpeak by 3.2%). Heartrate remained constant over the 12-month period and did not differ significantly (*p* > 0.05) between each test interval. Breathing reserve (BR%) improved over time, but was within a normal response at the peak exercise capacity [11,27].

Changes in VO2peak values over the course of 6 months of supervised training and after the end of supervision. T1 represents the start of the intervention, T3 represents the end of the monitored training period after 6 months, and T4 is the time point at the end of the non-monitored period. T4 also marks the end of the training program after 12 months. VO2peak values are expressed in mL/kg/min.

Percentage changes in VO2peak and Wpeak values over the course of 6 months of supervised training and after the end of supervision. T1 represents the start of the intervention, T3 represents the end of the monitored training period after 6 months, and T4 is the time point at the end of the non-monitored period. T4 also marks the end of the training program after 12 months. The VO2peak and Wpeak values are expressed as percentages of change relative to the starting point, T1, of the exercise training program.

## 4. Discussion

To the best of our knowledge, this is the first study to investigate the impact of a partially monitored, long-term exercise program in children with CF on exercise capacity, especially on VO2peak values. In addition, the effects on HPA during the monitoring period and after monitoring ended were also examined.

The results obtained in this study show there was a small beneficial effect on exercise capacity after 6 months of monitored exercise. However, during the monitored course of 6 months, the VO2peak values increased by 11%, and Wpeak values increased by 14%. Lung function, expressed as ppFEV1 and steps/day, remained nearly unchanged, and small effects on HPA were observed at the end of the six-month monitored exercise program.

A non-significant improvement was not observed in adolescent and adults with CF after the supervised exercise period [8,9]. In both studies, VO2peak values increased significantly after 6 months, whereas Wpeak values were only found to improve slightly. This change did not reach statistical significance. In absolute terms, however, in our study, the absolute changes in VO2peak (3.9 mL/kg/min) and Wpeak (0.4 W/kg) values were comparable with those changes in the studies mentioned above [8,9].

In contrast to the present study, a larger number of subjects participated in these studies, and the small number of participants studied here could partly explain the differences.

The aerobic training in children during an in-hospital stay produced a higher increase in VO2peak values by 21.6% or 7.31 mL/kg/min, respectively, which is higher than that in present study [19]. Compared to the present study, the children showed a more advanced disease severity and were admitted to the hospital due to exacerbation. Furthermore, the VO2peak values were higher in the present study (39.8 mL/kg/min vs. 33.8 mL/kg/min) and were within a normal range. A lower fitness level and lower lung function is associated with a greater training effect on VO2peak values, and this could explain the lower improvement in participants in VO2peak values in the present study [12].

The VO2peak and Wpeak values were within the normal range, which means that the children showed an age-appropriate exercise capacity at the beginning of the exercise program. In children and adults with CF, there seems to be a positive relationship between initial fitness level and improvement in maximal exercise capacity. Responsiveness to exercise training in pwCF depends on initial fitness level and lung function, with greater benefits observed in those people with lower fitness levels and lung function. Smaller improvements in VO2peak and Wpeak values could be explained by age-related normal and beneficial adaptations, which result in less trainability (Table 2) [12,31].

In comparison to short-term exercise programs, the responsiveness to training was lower in the present study [17,18,19]. In contrast to the home-based exercise program in the present study, which included contact with an exercise therapist every two weeks, the exercise sessions in short-term programs were carried out under supervision at a gym in a hospital at least once a week. In these studies, the effect of the exercise program was found to be larger than in home-based exercise programs, which was due to the participants’ closer contact with the physiotherapist [8,9,17,18].

However, it is somewhat difficult to compare different exercise programs because of the different training regimes used, the different ages of participants, the different durations, and the different aims of the studies.

In the present study, the positive effects did not persist after the end of the monitored training program. At the long-term follow-up at 6 months without monitoring, ppFEV1, the parameters of exercise capacity, steps/day, as well as HPA in moderate-to-intensive intensities and vigorous intensity declined.

Interestingly, the values for VO2peak and Wpeak were above the baseline values at the beginning of the monitored exercise program. These findings are in line with previous training studies from adolescents and adults with CF [8,9]. Therefore, it could be speculated that the children had fun exercising during the monitored program, and they were still motivated to do sports and exercise when they were not monitored. This could also explain the decrease in PA with sedentary intensity.

When monitoring was stopped, 8 out of the 14 participants terminated the program. It can, therefore, be suggested that in the majority of the children, close contact with the exercise therapist was of importance in maintaining motivation over a longer period. Close contact seems to be a guarantee for success in initiating beneficial training effects in children with CF [32,33].

Of note, a slight overall improvement in exercise capacity was observed without an increase in ppFEV1 values. Similar results were reported in earlier studies [8,9]. Hebestreit et al. discussed seasonal aspects as a reason for decreases in lung function [8].

The present exercise program was carried out throughout the whole year, and especially in winter, recurrent infections and climate conditions could have acted as barriers, which may have led to lower participation and adherence in exercise sessions, and a decrease in exercise capacity and lung function. Although PA and exercise at home were recommended in winter, e.g., with game consoles and active games alone, with friends, or family, seasonal aspects might explain the lower lung function values.

The present study failed to show an impact on PA in everyday life. According to the recommendation for exercise and HPA for people with CF [22], the children were sufficiently physically active at the baseline of the exercise training program, with at least 107 min of moderate-to-vigorous intensity and more than 10,000 steps per day. However, it is very difficult to increase time spent in PA in addition to attending school, medical treatment, and chest physiotherapy. A decline in PA of moderate-to-vigorous intensity was noticed after the 6-month, partially monitored exercise program, as well as a rise in physical activities with sedentary intensity (<1.5 METs) per day.

We recommended an increase in exercise activities by at least 30 min/week and a training intensity of 70% of the HRpeak value. These recommendations were lower than in comparable studies, in which patients exercised three times/week with a duration of 30–60 min and an intensity of about 65% of the VO2peak value [8,9,18].

It can be speculated that the lower threshold for achieving training effects in terms of the duration and intensity of training was too low to achieve major adaptations of aerobic cardio-respiratory performance. In addition, factors such as insufficient motivation or a lack of time due to disease-specific treatment, such as daily chest physiotherapy, school commitments, or seasonal effects (summer/winter), may have served as possible barriers, as described in other studies [5,8,9].

In addition, the children kept a training diary in which they recorded the physical activities they performed per day. The diaries were discussed with the children during telephone calls or clinical rounds at the hospital. It should be noted that some of the children did not list all of the days, and the effect of social desirability cannot be excluded in the description of training.

Therefore, it was difficult to acquire exact feedback on the physical sport activities in terms of the intensity and duration. To increase the motivation of the children to keep a diary, the diaries could be completed in digital form via social media.

In a paper published by our research group on CFmobil with adults with CF, we also observed an increase in the drop-out rate at the end of the supervised program [20]. To date, few studies have examined the effects of partially supervised exercise programs on participation [13]. We assume that close contact between exercise scientists or physiotherapists is an important factor, and is the basis to motivate pwCF to participate in exercise programs and to maintain regular physical activity. This seems to be an important requirement, especially for children with CF.

In the present work, a positive influence was shown between close supervision by experienced sports therapists and the children with CF, based on the improvements in VO2 and habitual physical activity (moderate-to-vigorous intensity) values. After the monitoring period stopped, no further increases, or for some aspects, a decrease, were detectable.

However, in the following 6 months, the participants either remained relatively stable at the previously achieved level, or the level was higher than that at the beginning of the exercise training program. Based on the results, it can be concluded that support in sport-specific questions for the children, and also for the parents, by sports therapists has a positive influence on the adherence to training programs [32,34].

As described above, many known barriers exist in pwCF which may prevent participation in regular physical sport activity [35]. The early education, especially of parents, but also of educators and teachers, by trained staff about the positive effects of regular physical activity is, therefore, of great importance. In this way, fears and concerns about possible negative effects on health can be allayed [33,36].

Especially in the era of CFTR modulators, this issue takes on greater importance, as pwCF have more opportunities to engage in physical activity due to improved lung function [37].

Some limitations of the study should be considered in the discussion of the results. The exercise program, as part of the CFmobil project, was offered to all patients older than 6 years of age in the participating CF centers as an additional treatment option. The pwCF participated voluntarily, and only children who were older than 10 years, taller than 120 cm, and who were being treated at the CF center in Bochum were eligible for CEPT. Thus, selection bias may have occurred, and the participants were not representative of the target groups treated in the other CF centers of the CHCR.

In further studies, children with a wide range of lung function and/or exercise tolerance should be motivated to participate in this treatment. The main limitation of this study was the low number of children included, and the lack of a control group. Thus, the results should be interpretated with caution. Further studies should include a larger number of participants and a control group, and should be designed as a randomized controlled trial. A high dropout rate could be found in the present study when monitoring stopped, and this aspect should be addressed in long-term studies, especially in children with CF.

## 5. Conclusions

In conclusion, this study demonstrated that a 6-month, partially monitored and individually tailored exercise training program increased the peak exercise capacity in children with CF with normal lung function and fitness levels. Improvements in physical activity were seen, but these changes were small. However, some beneficial effects persisted, whereas others decreased slightly when monitoring was stopped, indicating the necessity of close contact with a therapist to maintain the motivation to participate.

Tailored training programs considering the individual personal and environmental conditions and, additionally, the close contact between exercise therapists and pwCF is one of the basic requirements to motivate pwCF, especially children and adolescents, to participate in sports activities in the long term. Furthermore, parents should be educated at an early stage to explain the positive effects of physical activity on the development of children, as well as on disease-specific factors of CF.

Despite the limitations of this study, the results show beneficial effects, and highlight the need for the support of parents and therapists to motivate children to participate in regular exercise and PA.

## Figures and Tables

**Table 1:** Anthropometric characteristics, lung function, and steps/day of the CFmobil participants at baseline and end.

	Participants (n = 6)				
	Mean ± SD T1	Mean ± SD T3	Mean ± SD T4	Chi-Quadrat	*p*-Value
Age (years)	11.3 ± 3.3				
Height (cm)	143.6 ± 10.3	146.2 ± 13.0	147.6 ± 11.7 *	9.652	0.008
Weight (kg)	38.2 ± 10.0	38.5 ± 9.8	40.2 ± 10.8	4.261	0.119
BMI	18.2 ± 2.5	17.8 ± 2.4	18.0 ± 2.4	0.933	0.627
ppFEV1	102.5 ± 13.1	104.0 ± 8.7	92.3 ± 9.4 *	6.333	0.042
ppFVC	94.0 ± 13.7	95.3 ± 7.5	94.0 ± 11.1	0.333	0.846
Steps/day	10,791 ± 2103	10,835 ± 1072	10,298 ± 1905	1.600	0.449
Sedentary intensity (<1.5 METs) min/day	821.0 ± 116.7	724.0 ± 96.2	744.0 ± 107.9	2.800	0.247
Light intensity (1.5–3 METs) min/day	416.2 ± 71.6	442.0 ± 61.2	397.7 ± 75.6	0.737	0.692
Moderate-to-vigorous intensity (3–5.9 METs) min/day	107.3 ± 80.7	116.6 ± 66.3	97.3 ± 69.9	2.632	0.268
Vigorous intensity (>6 METs) min/day	16.3 ± 11.0	18.6 ± 14.3	16.8 ± 11.0	3.500	0.174

Abbreviations as described in text; T1 = baseline, T3 = after 6 months, T4 = end of program (12 months); Friedman two-way Analysis of Variance (ANOVA) by Ranks Test; between time points of measurement Wilcoxon Test * = *p* <0.05.

**Table 2:** Exercise capacity at baseline and end of the monitored exercise program, CFmobil.

	TG (n = 6)				
	Mean ± SD T1	Mean ± SD T3	Mean ± SD T4	Chi-Quadrat	*p*-Value
VO2peak (mL/kg/min)	39.5 ± 8.7	43.4 ± 9.3	41.2 ± 7.1	1.600	0.449
VO2peak (%pred)	88.6 ± 18.6	95.0 ± 18.2	91.0 ± 17.6	2.211	0.331
BR (%)	68.9 ± 17.0	74.3 ± 13.8	78.8 ± 11.8	0.400	0.819
Wpeak (Watt/kg)	2.9 ± 0.8	3.2 ± 0.6	3.1 ± 0.6	2.666	0.264
Wpeak(%pred)	95.5 ± 26.3	107.3 ± 22.8	104.0 ± 26.3	2.311	0.311
HFpeak (b/min)	184.8 ± 13.1	183.3 ± 14.3	183.0 ± 17.1	0.609	0.738
HFpeak (%/peak)	94.8 ± 6.7	94.0 ± 7.4	93.8 ± 8.8	0.609	0.738

Abbreviations as described in text; T1 = baseline, T3 = end of monitored exercise program (6 months), T4 = end of program (12 months); Friedman two-way Analysis of Variance (ANOVA) by Ranks Test.
